# Development and Validation of LC–MS/MS and IC–HRMS Methods for Highly Polar Pesticide Detection in Honeybees: A Multicenter Study for the Determination of Pesticides in Honeybees to Support Pollinators and Environmental Protection

**DOI:** 10.3390/jox15040095

**Published:** 2025-06-20

**Authors:** Tommaso Pacini, Emanuela Verdini, Serenella Orsini, Katia Russo, Tabita Mauti, Mara Gasparini, Marialuisa Borgia, Barbara Angelone, Teresa D’Amore, Ivan Pecorelli

**Affiliations:** 1 Chemistry Department, Istituto Zooprofilattico Sperimentale dell’Umbria e delle Marche “Togo Rosati”, Via G. Salvemini, 1, 06126 Perugia, Italy; e.verdini@izsum.it (E.V.); s.orsini@izsum.it (S.O.); i.pecorelli@izsum.it (I.P.); 2 Direzione Operativa Chimica, Istituto Zooprofilattico Sperimentale del Lazio e della Toscana “M. Aleandri”, Via Appia Nuova 1411, 00178 Rome, Italy; katia.russo@izslt.it (K.R.); tabita.mauti@izslt.it (T.M.); 3 Food and Feed Chemistry Department, Istituto Zooprofilattico Sperimentale della Lombardia e dell’Emilia-Romagna “Bruno Ubertini”, Via Bianchi, 9, 25124 Brescia, Italy; mara.gasparini@izsler.it (M.G.); marialuisa.borgia@izsler.it (M.B.); barbara.angelone@izsler.it (B.A.); 4 Laboratory of Preclinical and Translational Research, IRCCS CROB, Basilicata Oncology Referral Center, Rionero in Vulture, 85028 Naples, Italy; teresa.damore@crob.it; 5 Department of Pharmacy, University of Naples Federico II, 80131 Naples, Italy

**Keywords:** polar pesticides, honeybees, LC–MS/MS validation, IC–HRMS validation

## Abstract

The widespread use of agrochemicals raises concerns about environmental impacts, particularly on pollinators, such as bees, which serve as bioindicators of contamination. Developing methods to assess contamination risks in bioindicators supports regulatory frameworks, including EU regulations on the maximum residue limits (MRLs) for pesticides in food and the environment. This study presents the development and validation of two complementary analytical methods (LC–MS/MS and IC–HRMS) for highly polar pesticide (HPP) detection and quantification in bee matrices. Both methods were validated according to document SANTE/11312/2021 v2. LC–MS/MS was validated with a limit of quantification (LOQ) of 0.005 mg/kg for all the analytes. Repeatability at 0.005, 0.010, 0.020, and 0.100 mg/kg showed RSD_r_ from 1.6% to 19.7% and recoveries between 70% and 119%. Interlaboratory precision at 0.020 mg/kg across two labs showed RSD_R_ from 5.5% to 13.6%, with recoveries between 91% and 103%. The IC–HRMS method achieved LOQs of 0.01 mg/kg (glufosinate, N-acetyl glufosinate, MPPA, glyphosate, N-acetyl glyphosate, N-acetyl AMPA) and 0.1 mg/kg (fosetyl, phosphonic acid, AMPA), with mean recoveries in repeatability conditions from 84% to 114% and RSD_r_ from 2% to 14%. Intralaboratory precision showed mean recoveries from 87% to 119%, with RSD_wR_ values between 10% and 18%. These methods enable accurate monitoring of HPP contamination, supporting risk assessment and sustainable agriculture.

## 1. Introduction

The widespread use of agrochemicals has significantly increased in recent decades, raising concerns about their environmental and ecological impact. Among non-target organisms, pollinators such as honeybees are particularly susceptible to agrochemical exposure, threatening their survival and the vital ecosystem services they provide, particularly pollination. The European Parliament and other regulatory bodies have emphasized the urgent need to mitigate pesticide-related risks by promoting sustainable agricultural practices that protect environmental health, pollinator populations, and biodiversity [1]. Effective monitoring of pesticide contamination in pollinators is crucial for understanding environmental exposure and implementing strategies to safeguard these insects and the ecosystems they support.

Pesticide monitoring in pollinators must account for both the parent compounds applied in fields and their metabolites, which can form through environmental degradation and/or metabolic processes. While several studies have confirmed the presence of pesticide residues in pollinators, highlighting their role as effective bioindicators for environmental contamination, significant gaps still remain in the assessment of highly polar pesticides (HPPs), such as glyphosate, ethephon, fosetyl, and glufosinate, in bee populations [2,3]. Despite their high water solubility and extensive use in modern agriculture, these substances are often overlooked in pesticide monitoring programs aimed at assessing their presence in the environment, which traditionally focus on more lipophilic pesticides [4]. Therefore, the inclusion of polar pesticides in bioindicator studies is essential for comprehensive environmental risk assessment and the protection of pollinators.

Although validated methods for detecting highly polar pesticides in food, foodstuffs, and animal-origin products such as honey exist, there is a lack of established methodologies specifically tailored for their detection in pollinator insects. Previous studies demonstrated the effectiveness of high-performance liquid chromatography coupled with tandem mass spectrometry (HPLC–MS/MS) and ion chromatography coupled with high-resolution mass spectrometry (IC–HRMS) for analyzing polar pesticide residues in honey, fruit, and vegetables [5,6,7]. These studies have laid the groundwork for the development of more comprehensive pesticide monitoring approaches, but their applicability to unconventional substrates such as bees remains unexplored.

The complex nature of bee matrices presents unique analytical challenges, including the presence of biological substances such as wax and pollen, which can interfere with pesticide detection. Traditional multi-residue methods (e.g., QuEChERS) are unsuitable for the analysis of highly polar compounds due to their low recoveries and high matrix interferences. Consequently, adapting the existing techniques to the use in bee matrices requires substantial modifications to improve sensitivity, selectivity, and extraction efficiency [8]. The validation of novel HPLC–MS/MS and IC–HRMS methods for bee matrices is essential to bridge this analytical gap and enable robust, large-scale monitoring of polar pesticide contamination in pollinators.

For several polar pesticides, the legal definition of the maximum residue levels (MRLs) encompasses their metabolites, as these byproducts have a significant role in the risk assessment of the precursor in terms of toxicity and high yield of metabolization. For instance, regarding glyphosate, in 2019, the European Food Safety Authority (EFSA) conducted a review of its MRLs considering two different definitions for assessing animal-based commodities: one for monitoring purposes, including the sum of glyphosate, aminomethylphosphonic acid (AMPA), and N-acetyl-glyphosate, and another for risk assessment purposes, comprising the sum of glyphosate, AMPA, N-acetyl-glyphosate, and N-acetyl-AMPA, all expressed as glyphosate. However, due to insufficient data on the presence of glyphosate and its metabolites in animal-derived products, these MRLs remain provisional [9].

To address this gap, the EFSA emphasizes the need for confirmatory analytical methods for detecting glyphosate, AMPA, and N-acetyl-glyphosate in fat, kidneys, and liver, as well as methods for verifying the presence of AMPA and N-acetyl-glyphosate across all relevant matrices [9].

In this framework, member states conduct multiannual control programs (MACPs) for pesticide residues to ensure compliance with MRLs and assess consumer exposure to pesticide residues in both plant- and animal-based foods [10]. Regulation (EU) 2024/989, which governs the ongoing multi-annual control plan (2025–2027), mandates analyses of glyphosate and glufosinate ammonium in specific animal-derived products such as swine fat and cow milk (2025), bovine liver and poultry fat (2026), and chicken eggs and bovine fat (2027). Additionally, the plan mandates monitoring for other polar pesticides, such as ethephon and fosetyl, in plant-based foods [10].

In addition to the European legislation focusing on controlling highly polar pesticide residues in food, the EFSA and other regulatory bodies have established guidelines to monitor and manage these substances in environmental matrices such as water and soil. The EFSA has identified data gaps concerning the impact of water treatment processes on pesticide residues, emphasizing the need to assess the nature of these residues in treated water and their potential risks to human health and the environment [11]. Furthermore, the EFSA conducts risk assessments of the MRLs of active substances such as pesticides, considering their potential effects on non-target organisms, including bees [12].

Despite the considerable attention given to defining these limits, specific MRLs for polar pesticides in environmental indicators such as honeybees are not explicitly provided in the current EFSA guidelines. While the EFSA has established a specific protection goal (SPG) for honeybees, setting a 10% maximum acceptable colony size reduction following pesticide exposure, this does not equate to setting the MRLs for individual pesticides in bees, indirectly indicating a regulatory gap in directly linking residue levels to risk assessments for pollinators [13].

The development and validation of reliable analytical methods for polar pesticide monitoring in bees are of paramount importance for conducting wide-scale risk assessments of environmental contamination. The impact of pesticide exposure on bee health extends beyond direct toxicity, influencing critical physiological and behavioral aspects, such as gut microbiome composition, gene expression, foraging behavior, thermoregulation, and flight patterns [14,15,16]. Through the improvement of methods for the detection and quantification of polar pesticides in pollinators, researchers and regulatory bodies might better assess the risks associated with pesticide co-occurrence and inform policies aimed at reducing environmental contamination.

Implementing validated analytical methods also aligns with the principles of sustainable agriculture by promoting pesticide management strategies that minimize ecological harm. Reliable data on pesticide residue levels in pollinators can guide risk mitigation efforts, such as reducing pesticide application rates, implementing buffer zones, and promoting the use of less harmful alternatives. Additionally, such methods can support the enforcement of regulatory frameworks, including the European Union’s regulations on the maximum residue limits (MRLs) for pesticides in food and environmental matrices [17,18,19].

Recent data highlight the alarming consequences of pollinator decline linked to pesticide exposure and broader environmental contamination. The loss of pollinator biodiversity has far-reaching ecological and economic implications, affecting crop yields, food security, and ecosystem stability. Studies have demonstrated that pesticide-induced mortality and sublethal effects contribute to colony collapse disorder (CCD) and population declines in wild pollinators [20,21,22]. Additionally, the accumulation of pesticide residues in pollinators has been correlated with increased mortality rates, weakened immune responses, and reproductive impairments.

Given the critical role of pollinators in agricultural and natural ecosystems, it is imperative to implement comprehensive monitoring programs that encompass both traditional and highly polar pesticides.

The importance of developing new analytical methods for detecting various classes of pesticide residues in honeybees is evident from an in-depth review of the literature concerning the analysis of pesticides in honeybees, honey, and other hive products. In 2002, Morzycka developed a simple method based on solid-phase dispersion for quantifying 12 different non-polar pesticides in honeybees, using capillary GC coupled with NPD. The author noted that this method was suitable only for detecting low- and medium-polarity pesticides in honeybees [23].

In 2010, Kamel refined an analytical method for quantifying neonicotinoid pesticides and their metabolites in honeybees and bee products using LC–MS/MS, aiming to better understand their role in pollinator decline [24]. A major advancement came in 2015 with the work of Kiljanek et al., who developed a multi-residue method to detect 200 pesticides, such as neonicotinoids and pyrethroids, and their relative metabolites using GC–MS/MS and LC–MS/MS in combination with the QuEChERS and d-SPE techniques. However, this method also primarily targeted non-polar pesticides [25].

In 2017, Balsebre et al. developed a matrix solid-phase dispersion method combined with GC–FPD, GC–ECD, and GC–MS to detect 12 non-polar pesticides in honeybees from central Chile [26], and in 2018, Tosi et al. validated a method for the detection and quantification of 66 non-polar pesticides in pollen samples collected in Italy through UPLC–MS/MS [27]. Additional methods have since been developed for detecting pesticides, mainly neonicotinoids, in bee products such as bee pollen, beeswax, royal jelly, and honey, as reported by Valverde et al. in 2022 [28].

Starting in 2023, efforts to develop methods for detecting polar pesticides in beehive products emerged. Butovskaya et al. and Jesus et al. contributed to this area with studies on glyphosate and other polar pesticides. In the first study, the occurrence of glyphosate and other polar pesticides was assessed in honey, analyzing samples coming from two different Italian regions, while in the second one, a new method was validated to quantify highly polar anionic pesticides in beehive products (honey and pollen) [29,30].

That same year, Murcia-Morales et al. developed a method for detecting 74 non-polar pesticide residues using API strips, comparing the results with analyses conducted on honeybees [31]. More recently, in 2024, Ozols et al. conducted an occurrence study in Latvia, detecting and quantifying 21 non-polar pesticides using LC–MS/MS [32]. In 2025, Rampazzo et al. introduced a new IC–HRMS method for detecting glyphosate and glufosinate [33].

Despite the numerous studies focused on pesticide detection in honeybees and hive products, there remains a lack of validated methods for the simultaneous determination of multiple polar pesticides in the honeybee matrix.

Polar pesticides are known to be particularly susceptible to significant matrix effects. While several studies have investigated polar pesticides in animal-derived products and others have focused on non-polar pesticides in bees, there is currently a lack of research specifically addressing polar pesticides in insect matrices such as bees. Therefore, it is essential to validate and confirm the performance of the analytical method across a wide range of matrices to ensure its robustness and reliability in such complex and underexplored sample types.

The separation of polar pesticides using HPLC–MS/MS and IC–HRMS has historically presented significant challenges, primarily due to matrix co-extractives commonly found in foods of animal and plant origin [34,35]. This issue is particularly pronounced in the honeybee matrix, which is highly complex owing to its composition of proteins, lipids, enzymes, and chitin. These components can adversely affect analyte recovery and retention, as well as cause ion suppression or enhancement during mass spectrometric analysis. To mitigate these challenges, the separation parameters for both methods were optimized using a comprehensive, score-based approach, as detailed in our previously published work [36].

This study aims to overcome the lack in the literature, developing and validating two novel analytical methods, HPLC–MS/MS and IC–HRMS, for the detection of polar pesticide residues in bees. By addressing the current methodological gap, this research contributes to the advancement of environmental risk assessments and supports the transition toward more sustainable and pollinator-friendly agricultural practices. Ultimately, protecting pollinators from pesticide exposure is essential for maintaining biodiversity, ensuring food security, and preserving ecological balance.

## 2. Materials and Methods

The validation of the analytical methods was conducted by three different official laboratories [37], utilizing distinct techniques based on their available instrumentation. The Environmental Contaminants Laboratory at Istituto Zooprofilattico della Lombardia ed Emilia Romagna “Bruno Ubertini” (IZSLER), Brescia, Italy, performed analyses using ion chromatography (IC) combined with a high-resolution mass spectrometer (HRMS), while the Pesticides, Mycotoxins, and Plant Toxins Laboratory at Istituto Zooprofilattico dell’Umbria e delle Marche “Togo Rosati” (IZSUM), Perugia, Italy, along with the Environmental Contaminants Laboratory at Istituto Zooprofilattico del Lazio e della Toscana “M. Aleandri” (IZSLT), Rome, Italy, employed ultra-high-performance liquid chromatography (UHPLC) coupled with a triple quadrupole mass spectrometer (QqQ). Significant efforts were dedicated to optimizing chromatographic conditions, selecting suitable solvents for pesticide extraction, and refining sample purification procedures.

### 2.1. Chemicals and Reagents (LC + IC)

Reference standard solutions of AMPA, N-acetyl-AMPA, ethephon, glufosinate, glyphosate, N-acetyl-glyphosate, ethephon hydroxy (HEPA), 3-methylphosphonicpropionic acid (MPPA), and phosphonic acid, in water/acetonitrile (9:1 *v*/*v*) (1000 μg/mL); fosetyl-aluminum and N-acetyl-glufosinate (NAG) in water/acetonitrile (9:1 *v*/*v*) (100 μg/mL); and isotopically labelled internal standard (ILIS) solutions of AMPA 13C 15N, ethephon D4, glufosinate D3, N-acetyl-glyphosate D3, HEPA D4, MPPA D3, fosetyl-aluminum D15, NAG D3, and 18O3 phosphonic acid, in water/acetonitrile (9:1 *v*/*v*) (1000 g/mL), were purchased from Lab Instruments Srl (Castellana Grotte, Italy), HPC Standards GmbH (Cunnersdorf, Germany), Toronto Research Chemicals (Vaughan, ON, Canada), and Dr Ehrenstorfer (LGC Standards, Teddington, UK). The ILIS solution of glyphosate 2-13C, 15N (1000 μg/mL) in water was purchased from Cambridge Isotope Laboratories, Inc. (Tewksbury, MA, USA). Ethylenediaminetetraacetic acid disodium salt dihydrate (EDTA), 99–102% in purity, was obtained from Merck (Darmstadt, Germany). Methanol (MeOH; 99.9%), n-hexane (99%), formic acid (99%), and acetonitrile (99.9%) were obtained from Carlo Erba Reagents Srl (Milan, Italy). All solvents were of LC–MS or analytical grade. Unless otherwise specified, Milli-Q purified water (Millipore, Merck KgaA, Darmstadt, Germany) was used for sample preparation and analysis.

### 2.2. Samples (LC + IC)

**Figure 1:**
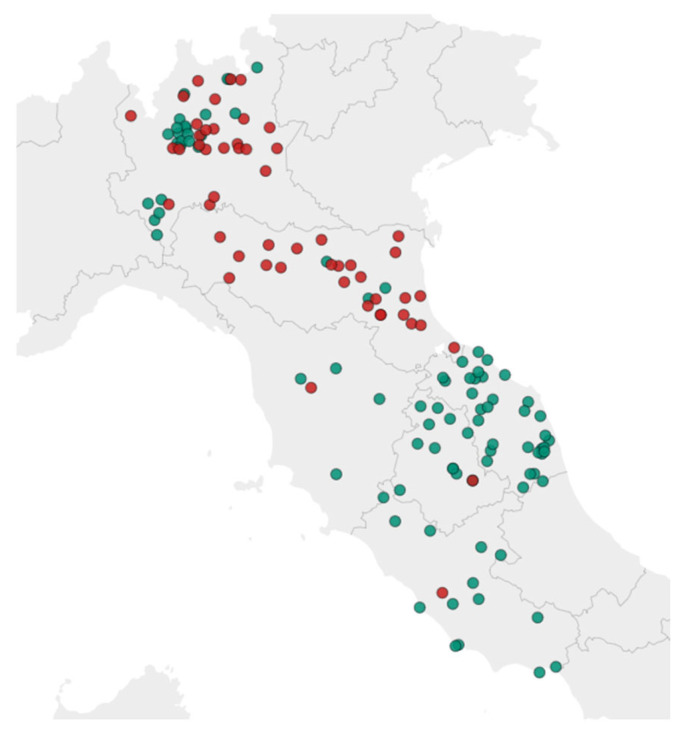
Sampling areas with elevated honeybee hive mortality rates (in red) and average honeybee hive mortality rate (in green).

A total of 314 bee samples, each weighing at least 10 g, were collected by several beekeepers across six Italian regions (Lombardy, Emilia Romagna, Umbria, Tuscany, Marche, and Lazio) from June to December 2023 on days with favorable weather conditions, typically during the morning hours to ensure consistent sampling. Among the samples collected, 22% originated from hives with a high number of dead or dying bees, while the remaining 78% came from healthy hives. For the validation studies, samples of live bees from apparently healthy hives were used. These samples had been previously analyzed to confirm the absence of detectable levels of the target analytes, in accordance with document SANTE/11312/2021 v2 [18]. Figure 1 illustrates the geographical distribution of the hives exhibiting higher mortality rates.

As shown in the map, regions with lower hive mortality are predominantly located in areas characterized by extensive agricultural practices, which may be associated with a reduced risk of pesticide exposure.

### 2.3. Analytical Methods

Two analytical methods, previously used in the laboratories for the identification and quantitation of polar pesticides in other kinds of matrices, were used as a reference for the development of the new ones:

1. Liquid chromatography coupled with tandem mass spectrometry (LC–MS/MS) for the detection of glyphosate, ethephon, glufosinate, fosetyl, and polar pesticide metabolites such as AMPA, N-acetyl-AMPA, HEPA, MPPA, and NAG in animal-derived products [6].
2. Ion chromatography coupled with high-resolution mass spectrometry (IC–HRMS) for the detection of the abovementioned compounds plus N-acetyl-glyphosate and phosphonic acid in vegetables, fruit, and honey [5].

#### 2.3.1. LC–MS/MS Method

The LC–MS/MS method for the detection and quantification of polar pesticides in animal-derived products was modified to be suitable for the bee matrix. After development, the method was fully validated to detect HPPs and their respective metabolites in honeybees, achieving sensitivity, recoveries (70–117%), repeatability (<20%), within-laboratory reproducibility (<20%), and experimental measurement uncertainty below 50% as required by the document SANTE/11312/2021 v2 criteria [18].

##### Reference Materials

The method provides the detection and quantification of 9 different target analytes (polar pesticides and their respective metabolites) with a LOQ of 0.005 mg/kg. The target analytes and isotopically labeled internal standards (ILIS) are reported in Table S1.

Three working solutions (WS) were prepared by appropriately diluting the stock and reference solutions to the following concentrations: (1) WS1: 1 μg/mL for AMPA, ethephon, fosetyl, glyphosate, HEPA, glufosinate, MPPA, NAG, and N-acetyl-AMPA; (2) WS2: 0.125 μg/mL for AMPA, ethephon, fosetyl, glyphosate, HEPA, glufosinate, MPPA, NAG, and N-acetyl-AMPA; (3) WS3: 0.01 μg/mL for AMPA, ethephon, fosetyl, glyphosate, HEPA, glufosinate, MPPA, NAG, and N-acetyl-AMPA. A mixed ILIS solution for spiking (WSIS1) was prepared by mixing commercial individual ILIS stock solutions to obtain a final concentration equal to 5 μg/mL for all the internal standards reported in Table S1. WSIS1 was diluted 40 times to prepare WSIS2, which was used for the matrix-matched calibration curve. Six-point matrix-matched calibration solutions, including isotopically labelled internal standards, were prepared by mixing appropriate volumes of WSIS2, WS2, and WS3 solutions with water and blank sample extract. The final volume of each calibrant solution was 500 μL.

##### Sample Preparation Protocol

The samples were stored at −20 °C until the analysis. Two grams of homogenized samples were weighed using a technical balance into polypropylene (PP) tubes with screw caps, and ILIS (20 µL of WSIS1) were added, along with 10 mL of methanol acidified with formic acid (1% *v*/*v*) and 8 mL of Milli-Q water. The samples were shaken for 5 min and then frozen at −20 °C for 10 min, followed by freezing at −80 °C for an additional 10 min. The frozen samples were centrifuged using a refrigerated ultracentrifuge at 15,000 RCF for 10 min at 0 °C. After centrifugation, a small portion of the organic layer, approximately 1.5 mL, was transferred to a plastic tube and then ultracentrifuged again at 15,000 RCF for an additional 10 min at 0 °C. Finally, the extract was filtered using a PTFE filter (13 mm, 0.22 μm) and diluted 1:1 with 250 μL of Milli-Q water in plastic vials, preparing it for LC–MS/MS analysis. Positive samples for validation purposes were prepared by spiking blank honeybee samples before the extraction procedure with 10 μL of WS1 and 20 μL of WSIS1. In each batch, one blank sample (without any addition of standard) was processed accordingly to prepare the six-point matrix-matched calibration curve.

##### Chromatographic and Spectrometric Conditions

The chromatographic method, previously developed and validated for different animal origin matrices, such as bovine fat and liver, chicken eggs, and cow milk [6], was modified to be suitable for honeybee matrices. The analysis was performed using a Triple Quadrupole AB Sciex 6500+ mass spectrometer (AB Sciex, Pte Ltd., Nijmegen, The Netherlands), coupled with a Nexera X2 UHPLC (IZSUM) and an Agilent 1290 HPLC (IZSLT). Chromatographic separation was achieved using an Anionic Polar Pesticide Column (2.1 × 10 mm, 5 μm), maintained at 50 °C. The mobile phase consisted of 0.9% formic acid in water (mobile phase A) and acetonitrile acidified with 0.9% formic acid (mobile phase B). The mobile phases’ composition started at 10% A, and the separation of HPPs was obtained by gradient as reported in Table S2. The total run time was 20 min, with a flow rate of 0.5 mL/min and an injection volume of 10 μL. The chromatographic protocol and mass spectrometer parameters are reported in Table S2; in Table S3, the electronic settings for each transition are indicated.

#### 2.3.2. IC–HRMS Method

The development and validation of the IC–HRMS method were carried out by modifying and adapting a previously validated method, developed in 2020, for the detection and quantification of polar pesticides in fruit, vegetables, and honey [5]. Honeybees, as an unconventional matrix, were studied and validated according to the criteria established in document SANTE/11312/2021 v2, confirming that the newly developed procedure is selective and robust [18].

##### Reference Materials

The method provides the detection and quantification of 10 different target analytes (polar pesticides and their respective metabolites) with different LOQs:

- LOQ = 0.01 mg/kg for glyphosate, N-acetyl-glyphosate, N-acetyl-AMPA, glufosinate, MPPA, and NAG.
- LOQ = 0.05 mg/kg for ethephon.
- LOQ = 0.10 mg/kg for fosetyl, phosphonic acid, and AMPA.

The target analytes, along with the respective measurement intervals and relative ILIS, are reported in Table S4.

Two working solutions (WS) in water/acetonitrile (9:1, *v*/*v*) were prepared by appropriately diluting the stock and reference solutions to the following concentrations: WS1: 1 μg/mL for glyphosate, NAG, N-acetyl AMPA, gluphosinate, N-acetyl glyphosinate, MPPA; 5 μg/mL for AMPA and ethephon and 10 μg/mL for fosetyl and phosphonic acid; WS2: 0.1 μg/mL for glyphosate, NAG, N-acetyl AMPA, gluphosinate, N-acetyl glyphosinate, MPPA; 0.5 μg/mL for AMPA and ethephon and 1 μg/mL for fosetyl and phosphonic acid. The ILIS for spiking in water/acetonitrile (9:1, *v*/*v*), ILIS1, was prepared by mixing commercial individual ILIS stock solutions to obtain a final concentration equal to 10 μg/mL for all the internal standards reported in Table S4. ILIS1 was diluted 10 times to prepare ILIS2 in water/acetonitrile (9:1, *v*/*v*), which was used for the matrix-matched calibration curve. Six-point matrix-matched calibration solutions, including isotopically labelled internal standards, were prepared by mixing appropriate volumes of the WS1, WS2, and ILIS2 solutions with the blank sample extract.

##### Sample Preparation Protocol

All the samples were stored at −60 °C for 24 h before homogenization. Afterward, one gram of the homogenized bee sample was extracted in 50 mL polypropylene tubes with 10 mL of a water:acetonitrile (1:1, *v*/*v*) solution containing 1% (*v*/*v*) formic acid and manually shaken. To ensure analytical accuracy, we monitored extraction efficiency and evaluated matrix effects; 20 μL of isotopically labeled internal standard solution ILIS1 in water/acetonitrile (9:1, *v*/*v*) at 10 µg/mL was added to each sample; the mixture was then mechanically shaken and centrifuged at 4000 RCF for 20 min at 4 °C. Subsequently, 750 μL of the supernatant were transferred to 15 mL polypropylene tubes and diluted with 3 mL of ultrapure water; this dilution was followed by the addition of hexane saturated with acetonitrile. The mixture was manually shaken to enhance phase separation and then centrifuged under the same conditions. Then, 1.5 mL of the lower aqueous phase was carefully collected and passed through a Sep-Pak Plus C18 cartridge and a 0.2 μm syringe PTFE filter in tandem. The resulting filtered solution was transferred to injection vials for final analysis.

##### Chromatographic and Mass Spectrometric Conditions

The ion chromatography method, previously developed and validated for different matrices, such as fruit, vegetables, and honey [5], has been modified and adapted for honeybee matrices. The analysis was performed using an Ionic Chromatograph IC 5000+ Dionex (Thermo Fisher Scientific, Waltham, Massachusetts, USA) coupled with a high-resolution mass spectrometer Q-Extractive Focus. The ion chromatography conditions are reported in Table S5.

The signals of the analytes in mass spectrometry coupled to the IC were acquired in the full mass mode, with negative ionization HESI. Additional parameters are reported in Table S6. For all the compounds, accurate mass and at least two MS2 fragment ions were detected with a relative mass error < 5 ppm. The inclusion list, in the instrumental method setting, contained retention time windows in which each precursor ion was expected, with their corresponding collision energies.

### 2.4. Validation Protocol

#### 2.4.1. LC–MS/MS Validation

The validation protocol was carried out following point G7 and Table 4 of document SANTE/11312/2021 v2 [18]. The following parameters were evaluated for validation purposes: linearity range, LOQ, recovery, precision under repeatability conditions as RSD_r_, and inter-laboratory precision in reproducibility conditions as RSD_R_. The same validation protocol was followed by both laboratories: IZSUM and IZSLT. The applied interlaboratory approach has wide-ranging use, highlighted in the literature as the preferred way to carry out the validation [38].

##### Calibration Curves and Linearity Ranges

Matrix-matched calibration curves were built as described in Section 2.3.1. The six calibration points were 0.0002, 0.0005, 0.0010, 0.0025, 0.0050, and 0.010 mg/kg. These ranges were selected considering a dilution factor of 20 applied during the extraction/purification process of all the samples. Calibration curves were obtained by plotting the response factor (RF) versus concentration (mg/kg) for each analyte. The RF was calculated as the ratio between the peak area of the analyte and the corresponding ILIS for all pesticides, except N-acetyl-AMPA, which was quantified without an isotopically labeled internal standard due to its unavailability. For linearity evaluation, calibration curves were injected on three different days spread over two weeks. The ordinary least squares (OLS) method, including the point (0;0), was used to determine the calibration function. For each calibration point, the back-calculated concentration (BCC) deviation, that is, the deviation of the calculated concentration by the calibration function (Cmeasured) from the true concentration (Ctrue), was evaluated using the formula:

$$Deviation=\frac{\left(Cmeasured-Ctrue\right)}{Ctrue}\times 100$$

According to document SANTE/11312/2021 v2 [18], the calibration curve was considered linear when, for each point, the BCC deviation did not exceed ±20%. Linearity ranges fulfilled the requirements for both IZSUM and IZSLT.

##### Recovery

Recovery experiments were independently performed by the two laboratories on four different spiking levels (0.005, 0.010, 0.020, and 0.100 mg/kg). The mass concentration was calculated using the following formula:

$$Cx\left(\frac{mg}{kg}\right)=\frac{\left(RF-a\right)}{b}\times DF/1000$$

where:

- RF = response factor (peak area ratio of the analyte/internal standard; peak area for N-acetyl-AMPA only).
- a = slope of the calibration curve (µg^− 1^).
- b = intercept of the calibration curve.
- DF = dilution factor (20).

The analytical parameters were evaluated by analyzing blank samples of bees fortified at four different concentration levels, as reported in Table S7. Level 1 was the estimated LOQ, level 2 was set at 2× LOQ, level 3—at 4 × LOQ, and level 4, the highest, was set at 20 × LOQ for all the target pesticides.

Validation experiments confirmed compliance with the performance criteria (recoveries between 70–120% and precision values lower than or equal to 20%). For inter-laboratory precision (RSD_R_), blank samples of homogenized bees were fortified at 0.020 mg/kg and analyzed by 3 different operators over 12 different days in two laboratories: Laboratory of Pesticides, Mycotoxins, and Plant Toxins (IZSUM) and the Environmental Contaminants Laboratory (IZSLT).

#### 2.4.2. IC–HRMS Validation

Instrumental linearity was assessed using six concentration levels in solvent-based solutions and matrix-matched standards. Specificity was evaluated by analyzing reagent blanks and blank samples to confirm no significant interferences near the characteristic retention times of each pesticide. Six fortified samples at two concentrations were analyzed to determine the limit of quantification (LOQ), recovery (trueness), and precision; matrix effects and linearity were assessed by comparing analytical response variations between solvent standard solutions and the matrix-matched calibration curve. Validation for IC–HRMS was conducted by IZSLER.

##### Calibration Curves and Linearity Ranges

Linearity was evaluated in matrix-matched standards using six calibration levels, obtained by mixing the appropriate amount of reference standards. The calibration points for each analyte are reported in Table S8. The analyses were performed using the response factor (RF) approach, which measures the relative mass response of an analyte compared to its ILIS; N-acetyl-AMPA was compared using ethephon hydroxy D4. AA and CA represent the area and amount of pesticide, respectively, while AIS and CIS correspond to the area and amount of the internal standard. The formula used is as follows:

$$RF=\frac{A_{A}\times C_{is}}{A_{IS}\times C_{A}}$$

RF was calculated for each calibration point and each pesticide. The average RF and its standard deviation were determined during the validation study. To assess RF acceptability, an internal criterion based on the standard deviation (% RSD) was established, requiring RF to remain at or below 20%.

During method development and validation, the feasibility of quantifying sample amounts using RF, derived from a matrix-matched calibration curve, was evaluated.

##### LOQ, Repeatability, and Within-Laboratory Reproducibility

The analytical parameters were evaluated through the analysis of blank bee samples fortified at two different concentration levels, as reported in Table S9. Level 1 was the estimated LOQ, level 2 was set at 10 × LOQ for all the target pesticides, with the exception of AMPA, for which the higher level was set at 5 × LOQ due to the method’s sensitivity for this analyte.

For the concentration levels defined above, validation experiments confirmed compliance with the performance criteria (recoveries within the 70–120% range and precision values lower than or equal to 20%) for all the pesticides. For the calculation of within-laboratory precision (RSD_wR_), blank samples of homogenized bees were fortified with each analyte at the LOQ level. A total of 18 replicates were considered through an ongoing precision evaluation.

##### Measurement Uncertainty

According to Appendix C of the SANTE document, for the three laboratories, the relative expanded measurement uncertainty (U’) was calculated, as requested by ISO/IEC 17025 [39]. The calculation was based on intra-laboratory validation/QC data as outlined in Appendix C, document SANTE/11312/2021 v2 [18]. The relative expanded measurement uncertainty was calculated through the application of a coverage factor (k = 2) (level of confidence approx. 95%) to the relative combined uncertainty (u’), determined by evaluating the relative standard deviation of within-laboratory reproducibility and the method bias.

The method and laboratory bias were calculated through recovery experiments conducted under an intra-laboratory reproducibility study, according to the following equation:

$$u^{\prime }\left(bias\right)=\sqrt{{RMS}_{\prime }\left(bias\right)}=\sqrt{{mean}_{bias}^{2}+{SD.P}_{bias}^{2}}$$

where:

mean_bias_ = mean of the corresponding bias

SD.P_bias_ = population standard deviation of the corresponding bias (calculated using the STDEV.P function in Excel, v.2505 build 16.0.18827.20102).

## 3. Results and Discussion

### 3.1. Analytical Method for Determination of Polar Pesticides in Bees Compared to the QuPPe-AO Method

As reported in Section 2.3, the reference analytical methods initially used were previously validated elsewhere for both LC/MS-MS [6], and IC-HRMS [5]. These two methods were developed for conventional food substrates, such as animal origin foods different from insects, as well as fruit, vegetables, and honey, based on the EURL-SRM QuPPe-AO-method v.3.3 [40]. However, some modifications were necessary to improve the recovery rate of polar pesticides extracted from this matrix, which is characterized by a high presence of wax and pollen, both of which can interfere with pesticide detection. To overcome this limitation, the extraction protocol was modified by introducing an additional ultracentrifugation step at low temperature (0 °C) at 15,000 RCF after transferring the extract into a 1.5 mL tube. To evaluate the optimal chromatographic conditions in LC–MS/MS for the separation, detection, and quantification of polar pesticides, three different chromatographic columns were tested: Hypercarb, Raptor Polar X, and Anionic Polar Pesticide. A score-based methodology was applied to determine the most suitable chromatographic column, with the Anionic Polar Pesticide column emerging as the optimal choice [36]. For IC-HRMS application, extraction with acidified water appeared to be the best option to ensure adequate compatibility with both the chromatographic columns and the gradient necessary for analyte elution. After extraction, an additional cleaning step using hexane saturated with acetonitrile was required to remove typical interferents from the material such as bees.

### 3.2. LC–MS/MS Method Performance Verification

A full in-house validation of the LC–MS/MS method was performed, including an inter-laboratory precision assessment through analysis conducted in two different laboratories: IZSUM and IZSLT. The following analytes were included: HEPA, MPPA, AMPA, ethephon, fosetyl, glufosinate, glyphosate, NAG, N-acetyl-AMPA. The validated parameters, according to SANTE/11312/2021 v2, included linearity ranges, recovery rates (%), repeatability (RSD_r_), and inter-laboratory precision (RSD_R_) [18].

#### 3.2.1. Linearity Ranges

The linearity ranges were independently confirmed by IZSUM and IZSLT using the BCC method, yielding fully satisfactory results for all the analyzed polar pesticides. Validation was performed at six levels: 0.0, 0.0002, 0.0005, 0.0010, 0.0025, 0.0050, and 0.0100 mg/kg, corresponding to a range of 0.004–0.200 mg/kg in honeybees, considering a dilution factor of 20 in the extraction method. For all spiking levels and in both laboratories, the deviation of BCC remained 20%. This satisfies the linearity criterion according to Table 4, point G of SANTE/11312/2021 v2 [18].

#### 3.2.2. Limit of Quantification (LOQ)

In the absence of specific MRLs for polar pesticides in honeybees, the LOQ, according to the SANTE regulation, was set to 0.005 mg/kg, between the second and the third point of the linearity range. This value was confirmed experimentally as the lowest spiked level meeting the identification and method performance criteria for recovery and precision. The LOQ for each analyte was independently validated by IZSUM and IZSLT [18].

#### 3.2.3. Repeatability (RSD_r_)

The evaluation of method precision under repeatability conditions, independently performed by IZSUM and IZSLT, demonstrated that for each analyte, the relative standard deviation (RSD) was below 20%, ranging between 1.6% and 19.7%, meeting the SANTE requirements at four different concentration levels (LOQ, 2× LOQ, 4× LOQ, and 20× LOQ) in six replicates. Additionally, recovery values ranged from 70% to 119%, fully complying with the SANTE acceptability criteria (point G, Table 4) [18]. Repeatability data are reported in Table 1.

#### 3.2.4. Inter-Laboratory Precision (RSD_R_)

The inter-laboratory reproducibility study was conducted on the honeybee samples spiked at 0.020 mg/kg in two different laboratories (IZSUM and IZSLT) by three different operators over four different days each (n = 12). The results confirmed the accuracy of the analytical method, with recovery values between 91% and 103% and RSD_R_ values between 5.5% and 13.6%, meeting the SANTE precision criteria (point G, Table 4) [18]. Inter-laboratory precision data are reported in Table 2.

The experimental relative expanded measurement uncertainties (U’) were below 50% for all the analyzed pesticides, thus remaining within the maximum expanded uncertainty limit set by the SANTE criteria [18].

**Figure 2:**
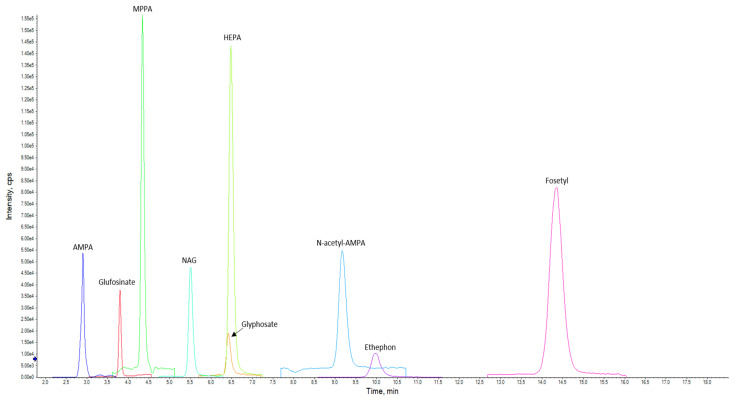
LC–MS/MS chromatogram of the matrix matched standard obtained with the present method. The spiking level was 0.100 mg/kg for each analyte (XIC).

A chromatogram showing the detected and quantified analytes with the LC–MS/MS method is reported in Figure 2.

### 3.3. IC–HRMS Method Performance Verification

A full in-house validation of the IC–HRMS method was performed by IZSLER, confirming compliance with all the required SANTE parameters, for the following analytes: MPPA, AMPA, ethephon, fosetyl, glufosinate, glyphosate, NAG, N-acetyl-glyphosate, N-acetyl-AMPA, and phosphonic acid. The validated parameters included linearity ranges, recovery rates (%), repeatability (RSD_r_), and within-laboratory reproducibility (RSD_wR_).

#### 3.3.1. Linearity Ranges

Linearity was confirmed by IZSLER using the BCC method, yielding fully satisfactory results for all the polar pesticides. The validation was performed at six levels, depending on the defined LOQ, as reported in Section 2.4.2:

- for fosetyl and phosphonic acid, the linearity was confirmed between 0.002 to 0.500 mg/kg;
- for ethephon and AMPA, the linearity was confirmed between 0.001 and 0.250 mg/kg;
- for glufosinate, N-acetyl-glufosinate, MPPA, glyphosate, N-acetyl-glyphosate, and N-acetyl AMPA, the linearity was confirmed between 0.0002 and 0.050 mg/kg.

The deviation of BCC, for all spiking levels, remained within ±20%, fulfilling the linearity criterion according to the SANTE document, as previously mentioned [18].

#### 3.3.2. Limit of Quantification (LOQ)

In the absence of specific MRLs for HPPs in honeybees, the LOQ, according to the SANTE regulation, was set at the lowest concentration satisfying the validation criteria reported in point G, Table 4 of the SANTE document [18]. After the confirmation of the accomplishment of the criteria designed for the highest concentration level of repeatability, recovery range between 70% and 120% and RSD_r_ less than or equal to 20%, the LOQ was defined by dividing the highest identified level by a factor of 10 and subsequently verifying the compliance with these two criteria at the lowest level experimentally for each analyte but AMPA, for which the LOQ was raised to 0.1 mg/kg.

#### 3.3.3. Repeatability (RSD_r_)

The evaluation of method precision in repeatability conditions performed by IZSLER revealed that for each considered analyte, the RSD_r_ was lower than 20%, in a range between 2.0% and 14.0%, meeting the SANTE requirements for two different concentration levels (LOQ and 10× LOQ), except for AMPA, which confirmed the requirements of the criteria at a concentration level of 5× LOQ in six different replicates. Additionally, fully satisfactory recovery percentage values were obtained, ranging from 84% to 114%, in compliance with the acceptability criteria established by the SANTE document, point G, Table 4 [18]. The repeatability data are reported in Table 3.

#### 3.3.4. Within-Laboratory Precision (RSD_wR_)

Data obtained from the within-laboratory reproducibility study of samples from honeybees, spiked at the LOQ, through 18 replicates from ongoing analyses, confirmed the accuracy of the analytical method. Recovery values ranged from 87% to 119%, with RSD_wR_ values between 10% and 18%, which is lower than 20% in any case, according to the precision criteria reported in point G, Table 4 of the SANTE document [18]. Within-laboratory precision data are reported in Table 4.

**Table 4:** Within-laboratory precision summary. The spiking level was at the LOQ for each analyte.

Analyte (LOQ) (n = 18)	IZSLER	
	Average Recovery %	RSD_wR_
AMPA	87	17.0
Etephon	103	18.0
Fosetyl	95	10.0
Glufosinate	94	11.0
Glyphosate	87	12.0
MPPA	97	10.0
N-acetyl-AMPA	119	16.0
NAG	93	10.0
N-acetyl-glyphosate	95	15.0
Phosphonic acid	98	12.0

Experimental relative expanded measurement uncertainties (U’) remained below 50%, in compliance with the SANTE requirements [18].

**Figure 3:**
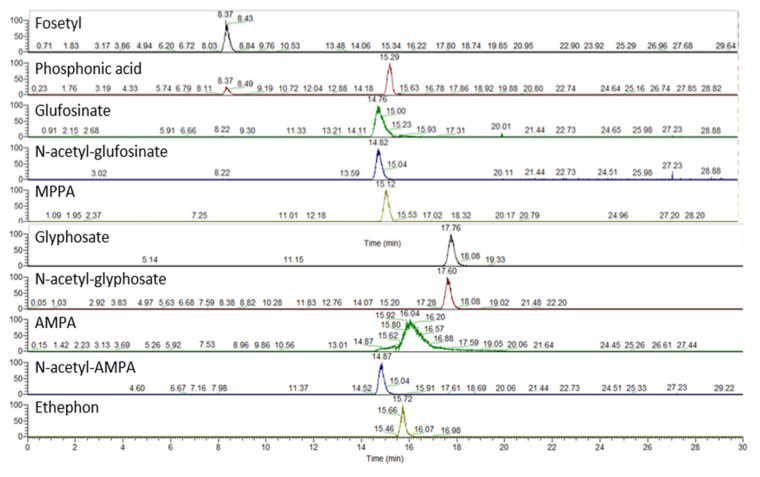
IC–HRMS chromatograms of the matrix-matched standard obtained with the present method. Spiking level 3 (see Table S8).

Chromatograms showing the detected and quantified analytes with the IC–HRMS method are reported in Figure 3.

#### 3.3.5. Measurement Uncertainty

To apply a default uncertainty of 50%, laboratories must demonstrate an experimental expanded MU (U’) value of ≤50%. The experimental U’ values based on intra-laboratory QC data for individual pesticides in honeybees are reported in Table 5.

## 4. Conclusions

In the present study, two novel and fully validated methods, LC–MS/MS and IC–HRMS, were successfully developed for the detection of HPPs in honeybees. These methods were validated through the collaborative efforts of three official laboratories of the Italian National Health System (Istituti Zooprofilattici Sperimentali), which are actively involved in the development of innovative analytical methods and consequent risk assessment to ensure food and environmental safety. The validation was carried out according to the criteria established by the SANTE guidelines, confirming the accuracy, sensitivity, and repeatability of both methods.

This study represents the first successful validation of the methods for detecting polar pesticides specifically in honeybees, and it establishes a fundamental step toward more comprehensive environmental monitoring. In the future, these validated methodologies could be useful as a reference for the detection of polar pesticides in other insect matrices and unconventional substrates, thus contributing significantly to food and environmental monitoring and risk assessment.

The LC–MS/MS and IC–HRMS methods are complementary due to the analytical challenges associated with detecting certain compounds. Phosphonic acid and N-acetyl-glyphosate, which are difficult to detect via LC–MS/MS, were effectively quantified through IC–HRMS, while HEPA and AMPA showed higher responsiveness and sensitivity in LC–MS/MS, with HEPA presenting high peak interferences in IC–HRMS. This complementary approach increases the robustness and reliability of the combined methods, enabling accurate and comprehensive detection of a wide range of polar pesticides.

Previously published data regarding the evaluation of different chromatographic columns [25] were used to choose the best-performing column and optimize the chromatographic performances for this unique matrix.

Furthermore, the exploitation of these innovative methods opens several future challenges and opportunities. One key challenge will be to establish new maximum residue limits (MRLs) for polar pesticides in honeybees and other pollinators, an initiative that could be undertaken by the EFSA and other international regulatory authorities. This effort could pave the way for adopting more sustainable agricultural practices that minimize pesticide exposure to pollinators and ultimately protect consumer health. Additionally, future research should focus on improving detection capabilities for a broader range of polar pesticides and their metabolites, as well as expanding these methods to cover other pollinator species. Addressing the ecological impact of polar pesticide contamination on bee health and pollinator populations remains a critical priority. Moreover, fostering collaboration between research institutions and regulatory bodies will be crucial in harmonizing methodologies and establishing new regulatory standards.

Finally, implementing these validated methods will significantly enhance environmental monitoring programs, facilitate data-driven risk assessments, and promote the adoption of healthier and more sustainable agricultural practices to protect pollinator health and environmental quality.

## Figures and Tables

**Table 1:** Repeatability data for each analyte at 4 levels of concentration, 0.005 mg/kg (LOQ), 0.010 mg/kg (2× LOQ), 0.020 mg/kg (4× LOQ), 0.100 mg/kg (20× LOQ).

Analyte	Level (mg/kg) (*n* = 6)	IZSUM		IZSLT	
		Average Recovery %	RSD_r_	Average Recovery %	RSD_r_
AMPA	0.005	93	4.0	102	10.6
	0.010	97	2.5	92	3.6
	0.020	101	5.0	88	6.4
	0.100	101	3.5	114	4.1
Ethephon	0.005	91	10.1	99	7.8
	0.010	93	3.4	110	3.0
	0.020	95	5.8	97	1.8
	0.100	95	4.3	115	1.6
Fosetyl	0.005	97	6.9	92	3.5
	0.010	87	5.2	98	6.0
	0.020	105	5.3	106	7.1
	0.100	101	3.3	106	3.8
Glufosinate	0.005	104	6.0	98	10.5
	0.010	96	8.8	92	6.6
	0.020	105	13.8	85	3.3
	0.100	99	4.3	98	6.3
Glyphosate	0.005	114	4.7	102	3.5
	0.010	102	4.6	115	5.7
	0.020	119	11.0	101	10.0
	0.100	98	10.8	108	3.4
HEPA	0.005	95	5.7	100	3.6
	0.010	84	6.8	111	4.2
	0.020	87	7.3	106	6.0
	0.100	102	4.4	117	1.8
MPPA	0.005	95	19.2	103	7.3
	0.010	99	3.6	94	3.1
	0.020	109	12.0	105	3.7
	0.100	104	6.6	111	2.3
N-acetyl-glufosinate	0.005	79	10.4	97	10.3
	0.010	95	6.7	74	14.8
	0.020	104	7.7	81	6.4
	0.100	104	3.4	99	3.0
N-acetyl-AMPA	0.005	79	7.7	93	19.7
	0.010	70	3.7	112	12.7
	0.020	91	3.9	108	4.2
	0.100	102	2.6	118	2.6

**Table 2:** Inter-laboratory precision summary. The spiking level was 0.020 mg/kg for each analyte.

Analyte (0.020 mg/kg)	Day	IZSUM		IZSLT		
		Operator 1	Operator 2	Operator 3	Mean	
		Recovery %	Recovery %	Recovery %	Average Recovery %	RSD_R_
AMPA	Day 1	107	89	77	93	7.2
	Day 2	87	100	87		
	Day 3	100	92	90		
	Day 4	102	102	93		
Ethephon	Day 1	84	100	98	91	6.9
	Day 2	88	95	94		
	Day 3	90	96	97		
	Day 4	71	80	98		
Fosetyl	Day 1	109	96	112	101	5.0
	Day 2	92	96	93		
	Day 3	97	100	106		
	Day 4	99	94	114		
Glufosinate	Day 1	101	92	85	93	7.5
	Day 2	93	101	89		
	Day 3	91	107	87		
	Day 4	99	90	80		
Glyphosate	Day 1	89	97	98	98	5.5
	Day 2	91	91	87		
	Day 3	102	103	116		
	Day 4	88	109	107		
HEPA	Day 1	99	107	111	103	6.6
	Day 2	90	80	99		
	Day 3	102	117	114		
	Day 4	96	114	109		
MPPA	Day 1	101	107	104	103	7.9
	Day 2	102	80	110		
	Day 3	102	117	109		
	Day 4	87	114	104		
N-acetyl-glufosinate	Day 1	104	96	82	91	9.8
	Day 2	90	94	76		
	Day 3	98	91	77		
	Day 4	97	105	81		
N-acetyl-AMPA	Day 1	92	95	115	94	13.6
	Day 2	67	86	105		
	Day 3	99	91	109		
	Day 4	78	76	113		

**Table 3:** Repeatability data for each analyte at 2 levels of concentration: LOQ and 5× LOQ for AMPA, LOQ and 10× LOQ for the other polar pesticides.

Analyte	Level (mg/kg) (*n* = 6)	IZSLER	
		Average Recovery %	RSD_r_
AMPA	0.10	93	6.0
	0.50	90	6.0
Ethephon	0.05	106	14.0
	0.50	98	10.0
Fosetyl	0.10	103	4.0
	1.00	108	3.0
Glufosinate	0.01	93	8.0
	0.10	98	3.0
Glyphosate	0.01	84	5.0
	0.10	93	4.0
MPPA	0.01	109	3.0
	0.10	114	4.0
N-acetyl-AMPA	0.01	100	7.0
	0.10	109	3.0
NAG	0.01	96	6.0
	0.10	110	2.0
N-acetyl-glyphosate	0.01	107	14.0
	0.10	85	6.0
Phosphonic acid	0.10	102	3.0
	1.00	100	2.0

**Table 5:** Experimental U’ for each laboratory.

	U’ (Expanded MU) (%)		
	IZSUM	IZSLT	IZSLER
AMPA	20	22	38
Ethephon	35	15	46
Fosetyl	15	18	49
Glufosinate	20	21	36
Glyphosate	22	9	37
HEPA	17	7	N.D.
MPPA	34	16	42
N-acetyl-AMPA	39	39	48
NAG	19	22	41
N-acetyl-glyphosate	N.D.	N.D.	35
Phosphonic Acid	N.D.	N.D.	41

## Data Availability

The original contributions presented in this study are included in the article/Supplementary Material. Further inquiries can be directed to the corresponding authors.

## Supplementary Materials

The following supporting information can be downloaded at: https://www.mdpi.com/article/10.3390/jox15040095/s1, Table S1: Target polar analytes in the standard mix and relative Isotopically Labeled Internal Standards; Table S2: Chromatographic LC conditions; Table S3: Mass Spectrometry Conditions; Table S4: Target polar analytes in the standard mix and relative Isotopically Labeled internal standards; Table S5: Ionic chromatography conditions; Table S6: Acquisition parameters for the mass spectrometry coupled to IC; Table S7: Validation Levels for Recovery, Repeatability and Within-Laboratory Reproducibility; Table S8: Matrix-matched points for linearity evaluation; Table S9: Validation Levels for Recovery, Repeatability and Within-Laboratory Reproducibility.
