# Supplementary material for: Development and Validation of LC–MS/MS and IC–HRMS Methods for Highly Polar Pesticide Detection in Honeybees: A Multicenter Study for the Determination of Pesticides in Honeybees to Support Pollinators and Environmental Protection
Source: J Xenobiot. 2025 Jun 20;15(4):95. doi: 10.3390/jox15040095 (PMC12387874; doi:10.3390/jox15040095)
Supplement: Supplementary file 1 [file jox-15-00095-s001.zip › jox-3647221-supplementary.pdf]

Supplementary Materials

Development and Validation of LC-MS/MS and IC-HRMS methods for highly polar pesticides detection in Honeybees: A multicenter study for the determination of pesticides in honeybees to support pollinators and environment protection.

Tommaso Pacini, Emanuela Verdini, Serenella Orsini, Katia Russo, Tabita Mauti, Mara Gasparini, Marialuisa Borgia, Barbara Angelone, Teresa D'Amore, Ivan Pecorelli

Target Polar Analyte	Measurement range (mg/kg)	ILIS
AMPA	0.005-0.200	AMPA ^{13}C ^{15}N
Ethephon	0.005-0.200	Ethephon D_4
Fosetyl	0.005-0.200	Fosetyl D_{15}
Glufosinate	0.005-0.200	Glufosinate D_3
Glyphosate	0.005-0.200	Glyphosate 2-^{13}C ^{15}N
HEPA	0.005-0.200	HEPA D_4
MPPA	0.005-0.200	MPPA D_3
N-acetyl-AMPA	0.005-0.200	N.D.
NAG	0.005-0.200	NAG D_3

Table S1. Target polar analytes in the standard mix and relative Isotopically Labeled Internal Standards.

Column	Anionic Polar Pesticide (2.1 x 50mm) 5 µm			
Pre-Column	Anionic Polar Pesticide VanGuard Cartridge (2.1 x 10 mm); 5 µm			
Acquisition Mode	Scheduled MRM			
Ionization Mode	ESI Negative			
CUR (psi)	40			
Collision Gas (CAD)	Medium			
IS (volts)	-4500			
TEM (°C)	550			
GS1 (psi)	60			
GS2 (psi)	60			
Injection Volume	10			
Cooler temperature (°C)	10			
Oven temperature (°C)	50			
Maximum temperature (°C)	90			
Pumping mode	Binary gradient			
Minimum Pressure (bar)	0			
Maximum Pressure (bar)	1000			
Pressure at t0 (bar)	65			
Chromatographic run Program				
Step	Time (min)	Flow (mL/min)	FM A (%)	FM B (%)
0	0	0.5	10.0	90.0
1	0.50	0.5	10.0	90.0
2	4.00	0.5	85.0	15.0
3	15.00	0.5	85.0	15.0
4	15.10	0.5	10.0	90.0
5	20.00	0.5	10.	90.0

Table S2. Chromatographic LC conditions.

Analyte Transition	Q1 mass (Da)	Q3 mass (Da)	RT (min)	MRM window (Sec)	EP	DP	CE	CXP
AMPA 1	110	63	3.05		-10	-34	-25	-9
AMPA 2	110	79	3.05		-10	-34	-29	-8
AMPA ^{13}C ^{15}N 1	112	63	3.05		-10	-34	-25	-9
Ethephon 1	143	107	9.9	180	-10	-19	-11	-9
Ethephon 2	145	107	9.9	180	-10	-19	-11	-9
Ethephon D_4	147	111	9.9	180	-10	-19	-11	-9
Fosetyl 1	109	81	14	200	-10	-24	-17	-9
Fosetyl 2	109	63	14	200	-10	-24	-40	-7
Fosetyl D_{15}	114	82	14	200	-10	-24	-17	-9
Glufosinate 1	179.9	63	3.8		-10	-30	-55	-6
Glufosinate 2	180	95	3.8		-10	-46	-23	-9
Glufosinate D_3	183	98	3.8		-10	-46	-23	-9
Glyphosate 1	167.8	62.9	6.45		-10	-30	-29	-10
Glyphosate 2	168	150	6.45		-10	-20	-15	-9
Glyphosate 2-^{13}C ^{15}N	170	63	6.45		-10	-20	-24	-9
HEPA 1	125	79	6.5		-10	-38	-33	-8
HEPA 2	125	95	6.5		-10	-38	-20	-5
HEPA D_4	129	79	6.5		-10	-38	-33	-8
MPPA 1	150.9	63	4.3		-10	-33	-43	-15
MPPA 2	150.9	71	4.3		-10	-33	-25	-20
MPPA D_3	154	136	4.3		-10	-33	-17	-8
N-acetyl-AMPA 1	151.9	63	9.05	180	-10	-30	-27	-10
N-acetyl-AMPA 2	151.9	110	9.05	180	-10	-30	-17	-10
N-acetyl-glufosinate 1	222	136	5.5		-10	-23	-27	-16
N-acetyl-glufosinate 2	222	63	5.5		-10	-23	-81	-9
N-acetyl-glufosinate D_3	225	63	5.5		-10	-23	-81	-9

Table S3. Mass Spectrometry Conditions.

Target Polar Analyte	Measurement Interval (mg/kg)	ILIS
AMPA	0.10 – 5.00	AMPA ^{13}C ^{15}N
Ethephon	0.05 – 5.00	Ethephon D_4
Fosetyl	0.10 – 10.0	Fosetyl D_{15}
Glufosinate	0.01 – 1.00	Glufosinate D_3
Glyphosate	0.01 – 1.00	Glyphosate 2-^{13}C ^{15}N
MPPA	0.01 – 1.00	MPPA D_3
N-acetyl-AMPA	0.01 – 1.00	Ethephon Hydroxy D_4
NAG	0.01 – 1.00	NAG D_3
N-acetyl-glyphosate	0.01 – 1.00	N-acetyl-glyphosate D_3
Phosphonic acid	0.10 – 10.0	Phosphonic acid $^{18}O_3$

Table S4. Target polar analytes in the standard mix and relative Isotopically Labeled internal standards.

Column		Dionex IonPAC™ AS19, 250 mm x 2 mm, 4 µm	
Pre-Column		Dionex IonPAC™ AG19, 50 mm x 2 mm, 4 µm	
Flow (mL/min)		0.25	
Column Temperature (°C)		40	
Samples Temperature (°C)		25	
Injection mode		Full loop	
Injection volume µL		50	
Acetonitrile auxiliary pump flow (mL/min)		0.20	
Suppression water flow (mL/min)		0.30	
Suppression voltage (mA)		90	
Mobile Phase		Gradient KOH	
Ionic Chromatographic run Program			
Step	Time (min)	KOH Concentration [mM]	Gradient type
0	0.00	5.00	
1	8.00	20.0	Linear
2	12.0	60.0	Linear
3	20.0	60.0	Isocratic
4	20.1	100	
5	25.0	100	Isocratic
6	25.1	5.00	
7	30.0	5.00	Isocratic
Divert Valve Parameters			
Time (min)		Flow from IC to	
0-20		Mass Spectrometer (Detector)	
20-30		Waste	

Table S5. Ionic chromatography conditions.

Analyte	Accurate Mass	Product Ion	Product Ion	Product Ion	Product Ion	CE	RT (min)
Fosetyl	109.0060	62.96417	78.95908			25	8.45
Fosetyl D_{15}	114.0374	62.96414				25	8.45
Phosphonic acid	80.9747	78.95909	62.96415			25	13.90
Phosphonic acid $^{18}O_3$	86.9874	84.97172				25	13.90
Ethephon	142.9670	78.95911	106.99019			10, 25	14.00
Ethephon D_4	146.9921	78.95912				25	14.00
Ethephon Hydroxy D_4	129.02303	78.95912				25	14.00
Glufosinate	180.0431	62.96420	94.99043	85.02953	136.0532	25	13.00
Glufosinate D_3	183.0620	62.96415				25	13.00
N-acetyl-glufosinate	222.0537	136.05331	62.96415	59.0138		25	13.20
N-acetyl-glufosinate D_3	225.0725	137.05945				25	13.20
MPPA	161.0166	62.96418	107.02669			25	13.80
MPPA D_3	154.0354	62.96415				25	13.80
Glyphosate	168.0067	62.96422	78.95898	80.97460		25	16.60
Glyphosate ^{13}C ^{15}N	171.0105	62.96415				25	16.60
AMPA	110.0113	62.96417	78.95909			25	13.00
AMPA ^{13}C ^{15}N	112.0016	78.95905	62.9641			25	13.00
N-acetyl-glyphosate	210.0173	62.96416	80.97460	78.95908		45	16.60
N-acetyl-glyphosate D_3	213.0361	62.96419				45	16.60
N-acetyl-AMPA	152.01182	62.96418	78.9591	110.0012		25	13.20

Table S6. Acquisition parameters for the mass spectrometry coupled to IC

LC-MS/MS Validation Levels (mg/kg)				
Analyte	Level 1	Level 2	Level 3	Level 4
AMPA	0.005	0.010	0.020	0.100
Ethephon	0.005	0.010	0.020	0.100
Fosetyl	0.005	0.010	0.020	0.100
Glufosinate	0.005	0.010	0.020	0.100
Glyphosate	0.005	0.010	0.020	0.100
HEPA	0.005	0.010	0.020	0.100
MPPA	0.005	0.010	0.020	0.100
N-acetyl-AMPA	0.005	0.010	0.020	0.100
NAG	0.005	0.010	0.020	0.100

Table S7. Validation Levels for Recovery, Repeatability and Within-Laboratory Reproducibility.

IC-HRMS Calibration Levels (mg/kg)						
**Analyte**	**Level 1**	**Level 2**	**Level 3**	**Level 4**	**Level 5**	**Level 6**
AMPA	0.001	0.0025	0.0125	0.050	0.125	0.250
Ethephon	0.001	0.0025	0.0125	0.050	0.125	0.250
Fosetyl	0.002	0.005	0.025	0.100	0.250	0.500
Glufosinate	0.0002	0.0005	0.0025	0.010	0.025	0.050
Glyphosate	0.0002	0.0005	0.0025	0.010	0.025	0.050
MPPA	0.0002	0.0005	0.0025	0.010	0.025	0.050
N-acetyl-AMPA	0.0002	0.0005	0.0025	0.010	0.025	0.050
NAG	0.0002	0.0005	0.0025	0.010	0.025	0.050
N-acetyl-glyphosate	0.0002	0.0005	0.0025	0.010	0.025	0.050
Phosphonic acid	0.002	0.005	0.025	0.100	0.250	0.500

Table S8. Matrix-matched points for linearity evaluation.

IC-HRMS Validation Levels (mg/kg)		
Analyte	Level 1	Level 2
AMPA	0.10	0.50
Ethephon	0.05	0.50
Fosetyl	0.10	1.00
Glufosinate	0.010	0.10
Glyphosate	0.010	0.10
MPPA	0.010	0.10
N-acetyl-AMPA	0.010	0.10
NAG	0.010	0.10
N-acetyl-glyphosate	0.010	0.10
Phosphonic acid	0.10	1.00

Table S9. Validation Levels for Recovery, Repeatability and Within-Laboratory Reproducibility.
